# Cases of Dropsical Ovaria, Removed by the Large Abdominal Section

**Published:** 1844-01

**Authors:** 


### ART. XIX.

*Cases of Dropsical Ovaria, removed by the large abdominal section.* By D. HENRY WALNE.—*London*, 1843. 8vo, pp. 66.

THIS pamphlet is a reprint from the Medical Gazette of the particulars of three cases, in which Mr. Walne removed dropsical ovaria with success. The history of the first of these cases is contained in the notice of Dr. Clay's paper, which appeared in our last Number; where will be found also such particulars, with reference to the second case, as Mr. Walne had communicated to the editor before the article went to press. We need, therefore, only mention, that the patient was fifty-seven years old; that her convalescence after the operation was retarded by an attack of phlegmasia dolens, but that, eventually, though slowly, she regained her health. The subject of the third operation was twenty years old, and unmarried; her recovery was not attended with any symptoms of really grave character. Want of space compels us to notice these cases very briefly; but it is only justice to Mr. Walne to say, that he appears to have watched his patients with unremitting care, and to have conducted their treatment very judiciously; to which the successful result of the operations was no doubt in great measure owing. The reports of their progress, too, are as full and explicit as could be desired.

Besides these cases, we may mention four others, in which a dropsical ovary has been extirpated with success. These operations were performed by Mr. Southam, Dr. Clay, Dr. F. Bird, and Mr. Lane. The particulars of the first three of these cases are in print;* a brief history of the last has been obligingly communicated to us by Mr. Lane. He tapped the ovarian cyst ten days before its removal; and was thus enabled to extract it through an incision reaching only from the umbilicus to the pubes, instead of from the sternum to the pubes, as in other cases in which the large section has been adopted. At the end of three weeks the ligature round the pedicle of the tumour came away; and Mr. Lane then considered the patient out of danger. Dr. F. Bird's case cannot with strict propriety be classed among those treated by the large operation; his mode of operating much more closely resembling that practised by Mr. Jeaffreson and Mr. Phillips, since he punctured the tumour, and after its contents had escaped, drew it through a small opening in the abdominal walls. The incision, however, was four inches long instead of an inch and a half, and differs in this more extensive wound of the peritoneal cavity from the minor operation, strictly so called.

Since the publication of his pamphlet, Mr. Walne has met with some unsuccessful cases, the particulars of which he will doubtless soon publish. In one of these cases, the operation was, we believe, left incomplete, but the person recovered; in another, the ovary was removed, but the patient died from the effects of the operation. Fatal cases have also occurred to Mr. A. Key,† to Mr. Greenhow,‡ and to Mr. B. Cooper.§

* Dr. F. Bird, in the Medical Gazette, Aug. 1843; Dr. Clay, in the Medical Times, Oct. 7, 1843; Mr. Southam, in the Medical Gazette, Nov. 17 and 24, 1843.

† Guy's Hospital Reports, Oct. 1843.

‡ Announced to be read at the Medico-Chirurgical Society

§ Mr. Cooper's case is likewise to be laid before the Medico-Chirurgical Society: but he kindly replied to some queries addressed to him by the editor. His answers enable us to

We therefore present the following summary, as being, to the best of our knowledge, a correct statement of the results of the large abdominal section.*

It has been attempted 37 times.

It has been actually performed 28 times; in 18 of these cases the patients recovered from the operation, in 10 they died: or deaths were to recoveries in a rather higher proportion than 1 to 2.

Of the remaining 9 cases, in which the operation was left incomplete, either because the tumours could not be removed, or because no tumour existed, 3 terminated fatally; in the other 6 the patients recovered.

We doubt much, indeed, whether the real results of this operation would present data so favorable as those we have just mentioned. We may feel pretty certain that we hear of all the cases in which this or any other unusually bold or bloody operation succeeds; but the confession of errors and failures, whose detail may promote the cause of truth but can never advance self-interest, requires a measure of moral courage to which, unfortunately, all are not equal. Nothing illustrates more strikingly the difference between the real and the apparent results of an operation, than the history of the Cæsarean section. Kayser, in his valuable dissertation, 'De eventu Sectionis Cæsareæ,' mentions that out of 341 cases on record, the recoveries are 127, or 37 per cent.; or nearly 2 recoveries in every 5 cases. If, however, we take the results afforded by the reports of lying-in-hospitals, in which concealment is impossible, and failure is necessarily as notorious as success, we find that of 67 cases only 14 recovered; or that recoveries were only 20 instead of 37 per cent., or 2 in every 10 cases instead of 2 in every 5. Yet it may be fairly assumed, that in these institutions patients would be more ably treated than elsewhere; that the best obstetric and surgical aid would be at hand, the most opportune time for the operation would be chosen, and the most appropriate subsequent treatment adopted. Everything would, therefore, lead us to expect that the results afforded by public institutions would greatly exceed all others in success; and we can account for the reverse being the case, only on the supposition that an immense number of the fatal cases of Cæsarean section which occur in private are never reported.

But even if we assume our present data to be correct, and that they do not at all overrate the success of ovariotomy, it may be fairly asked—Is an operation which is followed by the death of more than 1 in 3 of those in whom it is completed, a fit subject for boasting of as a triumph of art, as constituting an era in the annals of surgery? Is human life state that the person was thirty-two years of age; that the tumour removed weighed 32lbs. 4 oz.; and that the patient died of peritoneal inflammation, eight days after the operation.

* Mr. Walne's second case was not included in the statistical table in our last Number, because its particulars were not published at the time of the article going to press, and the details with which he kindly furnished the editor, and which we appended as a note at the end, were not sufficient to enable us to submit the case to the same sort of analysis as all the other cases in our tables were subjected to. We retain Dr. Clay's fatal cases where we placed them formerly, for the reasons stated at p. 392 of our October Number; and shall always regard the difficulty of diagnosis as to the nature, seat, and connexions of tumours supposed to be ovarian, as one of the grand objections to the operation. We are aware that these tables have provoked the spleen of more than one gentleman who thought that he had found a ready mode of carving his way to notoriety: we cannot help it—they are true.

so worthless, as to warrant the repetition of this operation, again and again, till, forsooth, we thus obtain more extended statistics? Mr. Key compares the condition of a patient labouring under ovarian dropsy with that of one suffering from stone, and suggests that the arguments for lithotomy in the one are analogous to those which justify ovariotomy in the other. We cannot admit the exactness of this analogy, seeing that in many cases of ovarian disease life is not very materially shortened, and, in a still larger proportion, is not rendered by any means intolerable; while the reverse of both these conditions notoriously exists in the calculous cases usually submitted to operation. But, if the analogy be granted, does it follow, because we practise an operation which is fatal to 1 in 7 * of those who submit to it, in order to relieve a patient from the stone, that we are therefore justified in performing an operation fatal to 1 in 3, in order to remove a diseased ovary? Besides, in lithotomy it is a thing almost unheard of, for the operation to be performed and no stone to be found in the bladder, or for its extraction to be impracticable; but in nearly 1 in 4 of the instances in which ovariotomy has been attempted, an analogous result has occurred. The difficulties attending the performance of lithotomy are so serious as to deter self-sufficient ignorance from attempting it; but, as Mr. Key says, in words very similar to our own, in the October number of this Review, and which stirred the bile of more than one of the doughty champions of ovariotomy: "The operation is of so simple a kind, requiring so little knowledge of anatomy, and so little skilful surgical manipulation, compared with what the other larger operations in surgery call for, that I need not dwell minutely on the steps of its performance;" or, as we should say, that there is imminent hazard of many women being destroyed, and the fair fame of the profession tarnished, by the recklessness of some whose heads are turned by a little notoriety, or even by the hope of attaining it; and who begin incontinently to dream of themselves as Hunters, Harveys, and Hallers.

Let us not be misunderstood: far be it from us to censure indiscriminately. We know that such men as Messrs. Key, Cooper, Lane, &c., can be influenced by no unworthy motives in performing such an operation; and we believe the best men may not only be justified but almost forced to perform this one, under peculiar circumstances. In reference to his own case, Mr. Key, after weighing the reasons for and against its performance, says: "Nevertheless, I thought it my duty to perform the operation;—a duty which every surgeon must occasionally feel to be a painful part of his profession, but from which a lower feeling must not induce him to shrink." Unfortunately, however, all have not treated this subject in the modest, self-forgetting, truth-loving spirit which becomes members of our noble profession.

We have endeavoured to show the results which have hitherto attended this operation, and have always stated the truth and the whole truth as nearly as we could arrive at it. We think that the often-repeated performance of this operation, while comparatively so little has been done towards facilitating our diagnosis of ovarian and uterine tumours, and while at the commencement of the operation it is a matter so very uncertain

* This estimate of the amount of mortality from lithotomy rather overstates than understates the fatality of the operation, as will be seen by reference to the very elaborate and valuable tables in Chapter III of Dr. Willis's work, 'On the Treatment of Stone in the Bladder.'

whether its completion will be practicable, is mere crass empiricism, wholly unworthy of the medical art. Each day, too, brings some fresh confirmation of our opinion; and in the Medical Gazette, for Dec. 9, 1843, the reader will find the particulars of a case that would justify language far stronger than we have ever used. A woman in ill health, and suffering from profuse periodical discharge from the uterus, was received into the Manchester Union hospital for the cure of an abdominal tumour. Mr. Heath and his colleagues, after repeated and careful examination, decided that the tumour was ovarian, and considered that the patient presented "a fair case for extirpation by the abdominal section." The abdomen was laid open by an incision, reaching from the ensiform cartilage to within an inch and a half of the symphysis pubis, when it was discovered that the uterus and not the ovary was the organ diseased. Mr. Heath, however, proceeded to remove it, and actually extirpated the entire uterus, except the cervix and about two inches of the body! The mass altogether weighed six pounds. The patient survived 17 hours after the performance of the operation. Respect for the manly honesty which Mr. Heath has shown in the early publication of this case, induces us to abstain from commenting on it; but indeed it comments on itself. We will only add, that we doubt much whether Mr. Heath would have ever had the hardihood to attempt the operation, still less would he have ventured to complete it as he did, if he had not been misled by the exaggerated and incorrect accounts that have been laid before the profession of the results of extirpation of the ovaria. And here we feel it our duty to animadvert, in the strongest terms, on the backwardness of those who, having so speedily published their successful cases, delay to publish their unsuccessful ones. We are unwilling to impute improper motives to these gentlemen; they may have good reasons for their silence of which we are ignorant; but the reasons must be strong indeed to justify a proceeding fraught with such momentous consequences to the profession, and to society at large.

We think, then, that a much more attentive investigation of ovarian disease and its treatment than has ever yet been made, is necessary, before we shall be able to predicate of certain cases that they are incurable by other means,—that this operation affords a reasonable prospect of their cure,—and that no modification of it will attain the same end. Till we can do this with tolerable certainty, the operation appears to us to be an unwarrantable proceeding. This opinion of ours may be wrong; we hold it simply because we have been led to it by a careful study of the subject, and will gladly retract it so soon as we find that we have been mistaken. In the meanwhile we would remind some gentlemen who seem to have forgotten it, that it is quite possible to arrive at conclusions different from their own, without the decision having been biassed by prejudice, or prompted by party-feeling. There is such a thing, sceptical though some may be with regard to its existence, as a sincere love of truth for its own sake; and we venture to express the belief that it always has been and always will be the characteristic of this Review.*

* Since this article was printed, the account of a second successful case by Dr. F. Bird has been published. The operation was of the intermediate kind, the length of the incision being rather less than six inches. At the date of the last report, (17th day from the operation,) the patient was convalescent. See 'Medical Times,' December 16.

